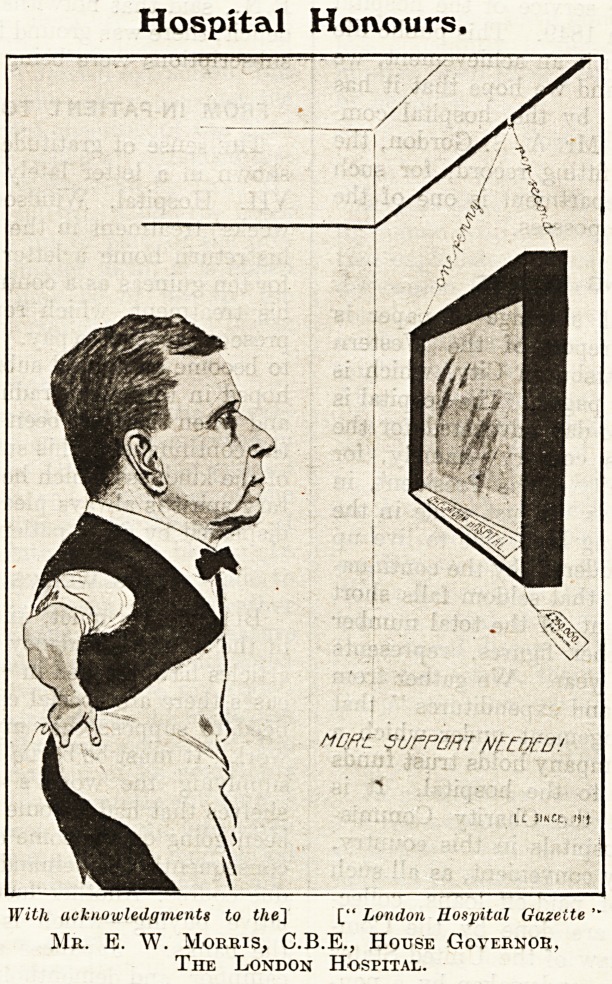# Hospital and Institutional News

**Published:** 1920-04-24

**Authors:** 


### HOSPITAL AND INSTITUTIONAL NEWS.

### THE HONOURS' LIST.

Two secretaries who were not mentioned in the leading article of last week were Mr. Arthur Griffiths, of the East Suffolk and Ipswich Hospital, who has received the O.B.E., and Mr. George Ruddle, General Superintendent of the Salford Royal Hospital, who had conferred upon him the membership of the same Order. Another interesting name in the list was that of Mr. F. A. Hocking, B.Sc., Pharmacist at the London Hospital. Mr. Hocking did excellent service for the Government on the Department of Overseas Trade, who had his advisory assistance by agreement with the London Hospital; he now receives the M.B.E., and had previously been appointed a Chevalier of the Order of the Crown by the King of the Belgians in recognition of his work on the Commission Internationale de Revitaillement.

### HEALTH MINISTRY'S NEW SECRETARY.

The Minister of Health has appointed Sir William Arthur Robinson, K.C.B., C.B.E., the Secretary of the Air Ministry, to be the First Secretary of the Ministry of Health, in succession to the late Sir Robert Morant. Sir Wm. Robinson, an Oxford man, has spent twenty-three years in the Civil Service. He was appointed Assistant Secretary to the Imperial Conference in 1907 and 1911, and Secretary of the Dominions Royal Commission in 1911-12. From 1912 until his appointment as Permanent Secretary to the Air Council in 1918 he was Assistant Secretary of the Office of Works.

### GOOD NEWS FROM GLASGOW.

After a sympathetic discussion, the committee appointed by the employees who subscribe to the voluntary hospitals of Glasgow has presented its recommendations. Though these have been referred to the workers for a mandate, and their decision will not be known until May, it is interesting to record the proposals. The principle is a contribution according to wages, not the customary flat rate; and the scale proposed is 1d. a week from wages under 30s. a week, 1½d. from wages between 30s. and 40s., 2d. from wages of 40s. to 60s., 3d. from 60s. to 90s., 4d. from 90s. to 120s., 5d. from 120s. to 140s., and 6d. from wages over 140s. per week. The scale, if adopted, is expected to yield £200,000 instead of the £87,000 at present raised from workmen's subscriptions. It is hoped also that at the same time the employers will increase their contributions. Mr. J. H. Turnbull, of the Western Infirmary, explained to the meeting at which the scale was reported that the workers at present contribute £66,000 towards the £100,000 required annually by the Royal Infirmary, £14,000 towards the £80,000 needed by the Western Infirmary, and £7,000 towards the £38,000 needed by the Victoria Infirmary. Several delegates cheerfully reported instances of the much increased contribution which a methodical collection had gained, and only a few expressed a preference for State support. Glasgow has been one of the stock examples of hospital indebtedness, and this initiative to mend matters there is very encouraging.

### NURSING HOME FOR THE NEW POOR.

Lady Astor, Dame Mary Scharlieb, and Lady Willoughby de Broke are amongst the signatories of an appeal for £8,000 to start a nursing home intended for the use of families of small means, or inelastic salaries, who are unable to pay the fees of an ordinary nursing home. It is intended that the staff will be composed of members of a Church of England Sisterhood with proper nursing qualifications, who are prepared to give their services gratuitously to this work, and that the home shall be run on ordinary nursing-home lines, with private rooms, and that the patients will be able to choose their own physician and surgeon. The home is to be called the St. Francis Home, and the Hon. Treasurer is Major Campbell, of 3 Southwell Gardens, S.W. 7. We suppose it is too late to attach this scheme to some existing hospital organisation. It seems a pity to start a new charity at the present time, especially when so many hospitals are contemplating action in the same direction and require funds for the necessary alterations and equipment.

### VALUE OF VACCINATION.

Bootle owes its thanks to Dr. W. Allen Daley, the medical officer of health, for the vigorous measures he has taken to stamp out the outbreak of smallpox in the borough. The infection was traced to a small vessel from a foreign port, and 400 contacts were established and kept under observation. The disease showed itself in one of the poorest parts of the town, and thither Dr. Daley went on his errand of education. Attended by the public vaccinator he harangued in the open air from his motor car crowds of men, women and children, pointing in plain language the moral of vaccination. The response to this unconventional campaign was remarkable, and over two hundred re-vaccinations were performed in forty-eight hours. His prompt effort not only saved the situation, but shows what may be done by one earnest individual to turn the tide of popular indifference to vaccination.

### ANTHRAX PREVENTION.

It is but fitting that Liverpool, which every year imports some ninety million pounds weight of wool classed as dangerous, should be selected as the site of the first of a series of trial stations designed to test the efficacy of disinfecion as a means of checking anthrax. The scheme has been prepared by Mr. G. Elmhirst Duckering, Secretary of the Departmental Committee on Anthrax (Disinfection), who is to be the executive officer in charge of the station. It is hoped to deal there with some six to eight million pounds of wool per annum, and the importers and manufacturers are giving their wholehearted support to the effort to neutralise the risks run by the operatives.

### SEVEN PAY WARDS FOR SURBITON.

The plan to extend Surbiton Hospital in commemoration of its jubilee, which will occur in October next, is noteworthy, because the chief feature will be seven new wards for paying patients. These should appeal to the residents, who belong to a class which will use them, and their support of the extension may do something to replace the help of the wealthy, many of whom are understood to have left the place. The sum asked for is £7,000, and Capt. Rigaud, the Chairman of the hospital committee, is confident that this will be raised, either by general appeal or methodical organisation. The plans of the extension, which have been drawn by Mr. Alfred Mason, include a new women's ward and additional rooms for nurses.

### A MEMORIAL TABLET.

We learn that a white marble memorial tablet to the late Lord Lister, with a medallion portrait in relief and bearing an inscription recording the fact that he was a student and house surgeon at University College Hospital between 1843 and 1852, has been presented to the hospital by the Lister Memorial Fund.

### ST. ANTHONY'S PIGS.

The ancient tradition regarding the historic pigs of St. Anthony has been revived in the annual report of King Edward VII. Hospital, Windsor: "It may possibly be of interest to note that the ancient tradition of St. Anthony's pigs still survives at the hospital. In far-distant times these creatures were a lucrative possession of the Dean and Chapter of Windsor, and, pastured where the hospital now stands, prospered exceedingly on the verge of the forest. (These pigs were subsequently transferred to London, and were for many years herded on the then unoccupied site near which the Bank of England stands to-day.) The pigs now in the hospital meadow may not, perhaps, be lineal descendants of those of St. Anthony, but they thrive equally well upon what would otherwise be wasted, and they are a tribute to the economy with which the institution is managed." Since the styes were first erected a couple of years ago the profit from the sale of the growing pigs has reached £214. The monks of the order of St. Anthony had a convent in Threadneedle Street, and they were so importunate for alms that they would threaten those who refused them with "St. Anthony's fire." Timid people were in the habit of presenting them with fat pigs to retain their goodwill. The pigs became numerous, and, as they were allowed to roam about for food, led to the proverb, "He will follow you like a St. Anthony's pig." Stow, however, states that when pigs were seized in the markets by the City officers as ill-fed or unwholesome the monks took possession of them, and, tying a bell about their necks, allowed them to stroll about on the dunghills until they became fit for food, when they were claimed by the convent. The monks had a free grammar school in Threadneedle Street, and the boys were called "Anthony's pigs" by the lads from St. Paul's School, who were thereupon dubbed as "Paul's pigeons" by their opponents.

### THE LATE SIR BLUNDELL MAPLE'S EXAMPLE.

It is remarkable that the hospitals which seem most badly off at present are with one or two exceptions in the City: the London Hospital, the Metropolitan Hospital, the Queen's Hospital for Children, Hackney Road. Unless we except University College Hospital, the most depressing statements seem to emanate from those nearest to the wealthiest centres of the capital. The juxtaposition of these names, however, suggests a reflection which we must continue to emphasise. University College Hospital owed in the past as much help to the late head of Maple's as to any one. He placed his resources largely at its disposal, and made its best interests his own. How far this happy alliance has been maintained is perhaps undetermined. But its existence should not be forgotten. The other hospitals named above are almost as close to other important firms as University College Hospital is to Maple's. Has the hospital made peculiar friends of its neighbours, the presiding geniuses of the Tottenham Court Road? Are their staffs organised in its support? Have they been asked with their employers to do so? We ask because we do not believe that Tottenham Court Road could be approached in vain. The same applies to friendly thoroughfares citywards of Whitechapel Road and Hackney. The moral is Dr. Johnson's, which applies to hospitals no less than to the middle-aged: "Keep your friendships in repair." We suspect that they have been neglected.

### SOME SUGGESTIONS FOR SALISBURY.

Salisbury Infirmary has now to be added to the growing list of institutions which have introduced the system of contributions by patients. Patients in a position to pay will be asked to contribute at the rate of half a guinea a week. The cost of their "keep" is computed at 15s. a week, and the actual cost to the institution per patient is about £3 a week. The Hon. Louis Greville reminded the Governors that these payments would bring only £1,500 a year. The real solution is to be found only in the methodical development of traditional sources of revenue; a system of regular contributions from employers and workpeople would do much to ease the strain. For instance, how much do the farmers of the Salisbury district contribute towards the upkeep of their infirmary? That is one field for inquiry. The Agricultural Labourers' Union, too, could probably be induced to give regular weekly support on the system which has been successful in South Wales and elsewhere.

### THE MINERS' GENEROSITY.

Mr. Charles Kenshole, chairman of the executive board of the Aberdare General Hospital, has presented a remarkable report. The unusual features were these—that notwithstanding the increase in the number of patients and in the cost of provisions and drugs, there was a surplus balance on the year of over £1,500, and the average cost per bed was reduced three guineas to £120 17s. 3d. The happy position is due in part measure to the ungrudging support of the miners, who found practically half of the institution's total ordinary income. They have now willingly increased their penny a week to threepence, in order to meet the cost of extensions. They and the hospital deserve to be congratulated.

### CHILD WELFARE IN BOMBAY.

Arrangements in connection with the Children's Welfare Work Exhibition to be held in Bombay soon are being vigorously pushed forward. The appeal for help is meeting with a good response. Over a lakh of rupees has been collected so far, including two anonymous donations of Rs.25,000 and Rs.20,000 respectively, that is, a total of about ten thousand pounds sterling.

### A MATERNITY HOSPITAL FOR YORK.

The York City Council is to establish a maternity hospital in connection with the York Dispensary. The Health Committee has an option to purchase Acomb Hall, with about fifty-three acres of land just outside the city. The hall is commodious, stands high, and has accommodation for some sixty beds. The Dispensary Directors offer to contribute £1,500 and state that an annexe in a central position will be necessary, in which a sister and one or two probationers may live, from which maternity cases may be visited at their homes, and an ambulance to take patients and nurses to and from the hospital. The Health Committee recommends these proposals.

### A POINT FOR THE BALANCE SHEET.

The Royal Hants County Hospital, Winchester, is considering the question of contributions from patients, and the building of a pay-ward. These suggestions, which spring from the increased cost of maintenance, are made with another which deserves notice. The accounts lately presented by Mr. L. B. Keyser, the hon. treasurer, show, he points out, that the County Council grant toward the maintenance of the V.D. Ward does not balance the expenditure. It is therefore urged that the County Council should make a more adequate grant. We do not know how rigidly these maintenance grants are compared elsewhere with the cost of the work supposed to be supported by them. We would, therefore, remark that the cost of the work done for these cases, for school-children, and other special classes of patients, subsidised by public bodies, should be carefully recorded in the accounts. In all these cases where the grant is less than the cost of maintenance, the public authority should be approached. We conclude that there is no such body in England which would wish to have its work done at the expense of the voluntary hospitals. The matter is worth careful attention.

### Hospital Honours.

*With acknowledgments to the* [*"London Hospital Gazette"*

Mr. E. W. Morris, C.B.E., House Governor, The London Hospital.

### THE FUTURE OF THE FOUNDLING HOSPITAL.

The announcement that the Foundling Hospital, so rich in associations with Handel, Hogarth, Dickens, may soon remove from its historic quarters cannot be received without regret. The site is worth anything from half a million to a million; fresh air is desirable for the children, and so on. This "hospital for exposed and deserted children," which was founded in 1739, has a history as curious and interesting as that of any institution in London. The foundling proper and the child whose mother wished to desert it, were equally welcome in the basket for babies which used to hang outside the gate. The hospital is rich in works of art or of artistic interest. Handel's organ, pictures by Reynolds, Hogarth and Gainsborough adorn its walls. The chapel has long been a place of pilgrimage to visitors on Sundays. If the children have to go, is the institution, the building, to become the prey of the speculative builder? Can Mr. R. H. Nichols, the secretary, reassure us on any point at all in this matter? With the departure of the children the heart of the place will have gone, but the bricks and stones should be dear to Londoners as well.

### EXTENSION IN STRATFORD.

The committee of the Stratford-on-Avon Hospital has decided to build a new out-patient department, improve the offices, and thereby increase the number of beds from thirty-one to fifty-one by appropriating some of the space set free to the wards. The cost of these changes is expected to be £26,000, and there is no immediate prospect of providing beds for paying patients.

### IN HOSPITAL LIFE SINCE 1849.

The chairman of the Royal Isle of Wight County Hospital, Mr. B. O. Cochrane, lately referred to what is surely an unrivalled record of service in hospital life. Among recent retirements and resignations Mr. Cochrane mentioned that of "a very old and very much respected servant," Mr. George Dash, who has been in the service of the hospital ever since it was founded in 1849. This public life of three-score years and ten is an achievement, we imagine, without parallel, and we hope that it has been generously recognised by the hospital committee. We hope also that Mr. A. S. Gordon, the secretary, will keep some fitting record, for such length of service in any department is one of the best traditions a hospital can possess.

### AN INTERESTING REPORT.

No sign of the national shortage of paper is betrayed by the annual report of the Western Pennsylvania Hospital, Pittsburgh City, which is a substantial volume of 109 pages. The hospital is on just such lines as are to-day advocated for the medical institutions of this country—namely, for all classes of the community. The President, in his report, says that he feels "a just pride in the hospital management, having been able to live up to the high ideals of its founders" by the continuation of the charity feature, that seldom falls short of 40 per cent. to 50 per cent. of the total number of the patients, or, in other figures, represents about 70,000 free days in a year. We gather from the accounts of "receipts and expenditures" that there is some sort of arrangement under which a Fidelity, Title and Trust Company holds trust funds and pays over the income to the hospital. It is not generally known that the Charity Commissioners will do this for hospitals in this country, and the arrangement is very convenient, as all such services as reinvestment of paid-off loans, collection of income tax, etc., are done by the Commissioners. Possibly the law of the United States allows these functions to be undertaken by a non-official body.

### NEWCASTLE ORTHOPÆDIC HOSPITAL.

A building feat, and all that that implies in other directions, has been achieved in connection with the Newcastle Orthopædic Hospital, which may be regarded as an extension or outpost of the Newcastle Infirmary. In 1917 the idea was formed, and now there are 500 beds which, Mr. C. Irwin lately remarked, were in permanent, not temporary, buildings. The cost of the scheme has been £152,000, of which £130,000 has been raised. The buildings were begun in May 1918, some were ready for occupation within eighteen months, and the whole will have been completed within two years. A formal opening has yet to be arranged, for it is felt that the most should be made of the occasion, which reflects great credit on all concerned.

### SALARIES AT LINCOLN.

It has been decided to increase the salaries of the secretary, matron, house-surgeon, and assistant house-surgeon at Lincoln County Hospital. The secretary's is to be raised from £400 to £600; the matron's from £150 to £220, and eventually to £250; the house-surgeon's and assistant house-surgeon's by instalments to £300 and to £200 respectively. The chairman, Capt. H. I. W. Nevill, R.N., said that notwithstanding an increase in the deficit, there was ground for confidence, because new subscriptions were being received.

### FROM IN-PATIENT TO ANNUAL SUBSCRIBER.

The sense of gratitude among patients is happily shown in a letter lately received at King Edward VII. Hospital, Windsor. After nearly twelve weeks' treatment in the wards, a patient wrote on his return home a letter accompanied by a cheque for ten guineas as a contribution towards the cost of his treatment, which represented the limit of his present capacity to pay. He added that he intended to become an annual subscriber of five guineas, and hoped in this way gradually to discharge his debt, and when that had been accomplished, to show, by the continuance of his subscription, his appreciation of the kindness which he had received. The voluntary spirit is always pleasant to see, not least when displayed by an in-patient.

### THIS WEEK'S DRUG MARKET.

Business is quiet, and there is a decided break in the upward tendency of prices. A number of articles have receded in value, but as in most of the cases there are special causes, there does not seem need to suppose that mysterious influences are at work. It must be borne in mind that the process of supplying the world's demand for drugs to fill shelves that had become empty during the war has been going on for some considerable time, and that consequently the demand will become less urgent in due course. Another factor is the decline in speculative buying—which is not being encouraged by the banks. Japanese articles, such as menthol, camphor, and dementholised peppermint oil, are all cheaper, mainly in consequence of the desire of Japan to turn her goods into money. Potassium bromide is offered at irregular rates, but the price tendency is upwards at the moment. Citric acid is still scarce, and the high price is firmly maintained. Buyers of cod-liver oil are still holding off in the hope of lower prices. Cottonseed oil is cheaper, and the same applies to linseed oil. The value of opium is firmly maintained. Liquorice juice is offered at lower rates. There is no substantial change in quotations for synthetic drugs. Calumba root is again lower in price. Generally speaking, buyers are more cautious in doing business.

## Figures and Tables

**Figure f1:**